# Electronic patient portal activation and outcomes among pediatric patients with asthma

**DOI:** 10.1016/j.jaip.2023.03.019

**Published:** 2023-03-21

**Authors:** Dariush Kafashzadeh, Kaitlin Hall, Cenan Pirani, Peter G. Szilagyi, Lucia Chen, Mindy K. Ross

**Affiliations:** a Department of Pediatrics, David Geffen School of Medicine, University of California Los Angeles, Los Angeles, Calif; b Department of Medicine, David Geffen School of Medicine, University of California Los Angeles, Los Angeles, Calif

With a focus on shared decision-making, more health systems are using patient portals, an online platform connected to the electronic health record (EHR) that allows for more patient engagement.^1–3^ Portals are helpful for chronic conditions such as asthma,^4,5^ yet little is known about portal users among pediatric patients with asthma or the relationship between portal use and clinical outcomes;^3^ similarly, little is known about adult portal users with asthma, but it has been shown that primary Spanish-speaking patients were less likely to use the portal than primary English speakers.^6^ In addition, the recent coronavirus disease 2019 (COVID-19) pandemic made in-person visits more challenging, raising the potential value of the portal for asthma management. The objectives of this study were to (1) compare characteristics of pediatric patients with asthma with and without an activated portal, (2) assess the relationship of portal use to asthma-related emergency department (ED) visits and hospitalizations, and (3) evaluate demographic and outcome changes after COVID-19.

We conducted a cross-sectional data analysis of portal use from March 19, 2019, to March 19, 2020 (the year before the first city-wide stay-at-home order) and from March 19, 2021, to March 19, 2022 (after COVID-19 vaccines became available and when health care utilization began normalizing) within the University of California, Los Angeles Health System. We first identified pediatric patients (<18 years) with a diagnosis of asthma ever with an outpatient primary care (pediatrics, medicine-pediatrics, and family medicine) or specialty (pulmonology and allergy/immunology) visit within the specified time periods. We then compared demographics between those with and without an activated portal (defined as having logged into the portal at least once) between the aforementioned time periods using χ^2^ and *t* tests. We next assessed ED visits and hospitalizations for asthma (primary, admission, or discharge diagnosis) during both time periods using χ^2^ tests. Outcome events occurred after portal activation. Univariate and multivariate logistic regression estimated unadjusted and adjusted odds ratios (ORs). A 2-way interaction term between portal activation and language was used to estimate ORs for language subgroups. Analyses were conducted in SAS v9.4 (SAS Institute, Cary, NC). Two-tailed tests were used, and *P* < .05 was considered statistically significant.

Before COVID-19, 80% of our cohort had an activated portal. These users were more likely to be White, have private insurance, preferred English language, be female, and have a lower (ie, less socially vulnerable) Social Vulnerability Index, which refers to potential negative community effects caused by external stresses (Table E1 and text, available in this article’s Online Repository at www.jaci-inpractice.org).^7^

During the post–COVID-19 vaccine period, 92% of our cohort had an activated portal status (Table E1, available in this article’s Online Repository at www.jaci-inpractice.org). Other than sex and age distribution, the statistically significant pre–COVID-19 demographic differences between the activated and nonactivated groups persisted. In the second period, there was an increase in most measured uses of the portal (reading physician messages and reviewing medications were most frequent) (Table E2, available in this article’s Online Repository at www.jaci-inpractice.org).

Before COVID-19, there was no difference in rates of ED visits for asthma, but nonactivated patients were more likely to be hospitalized for asthma than their activated counterparts (6.9% vs 4.9%, *P* = .002) (Figure 1). In the postvaccine period, nonactivated patients had a higher proportion and odds of ED visits (11.7% vs 7.7%, *P* < .001; unadjusted OR [uOR] = 0.62, *P* < .001) and hospital admissions (9% vs 3.8%, *P* < .001; uOR = 0.39, *P* < .001) for asthma exacerbations than portal-activated patients (Figure 1 and Table I). Significantly lower odds of ED or hospital visits for portal-activated patients persisted when controlling for other demographics. However, when stratified by language, this association was not significant for those with preferred language of Spanish (Table I).

Our results show that even with high baseline rates of portal activation among pediatric patients with asthma at our institution, portal activation increased further during the pandemic. However, racial and ethnic groups made marginalized including those preferring Spanish and more social vulnerability were less likely to use the portal even after the pandemic. This is consistent with other studies demonstrating that those historically marginalized face many barriers to accessing care including less awareness of the portal, less instruction, no or limited portal interface in their preferred language, and less digital literacy.^8^

Reasons for less ED visits and hospitalizations in those with activated portals are unclear. Perhaps patients could more easily contact their practitioner for remotely managed care to avoid an ED visit or be promptly directed to the ED, preventing worsening of symptoms to avoid hospitalization. An activated portal itself may have influenced behavior. Favorable outcomes did not appear to extend to those whose preferred language was Spanish (there was no asthma severity difference between these groups in *post hoc* analysis). One possibility is that while our portal has some Spanish translation tools, fully using the portal in Spanish for communication with practitioners can remain difficult.

Study limitations include the following: this was a secondary EHR data analysis from one academic center, and our patient population may not represent the broader pediatric asthma population, particularly given the high portal activation rates even before the pandemic. In contrast, in a 2021 study of pediatric portal use in Iowa, portal activation was approximately 40% in those previously hospitalized.^3,9^ In addition, some variables in our model (eg, asthma severity, smoking, race, etc) may not be completely or inaccurately captured because of unspecified and limited answer choices. We included only Spanish as a non-English language because our portal has only modifications for Spanish and numbers of those who preferred other languages were low. Our postvaccine period encompasses constant changes in terms of mask wearing, societal restrictions, and multiple subvariant waves, which are difficult to capture and may impact portal use and asthma-related ED visits and hospitalizations. Lastly, although our analysis showed statistically significant associations between portal activation and outcomes, they do not show causation. Further investigation into comorbidities (such as body mass index) and nuances of portal use is warranted.

Our data suggest that electronic portals hold promise in improving asthma-related clinical outcomes, although not necessarily for all groups. Further efforts to increase portal activation in historically marginalized groups who may face greater barriers to activation are some of the next steps in increasing portal use and improving asthma clinical outcomes.

## Supplementary Material

1

## Figures and Tables

**FIGURE 1.**
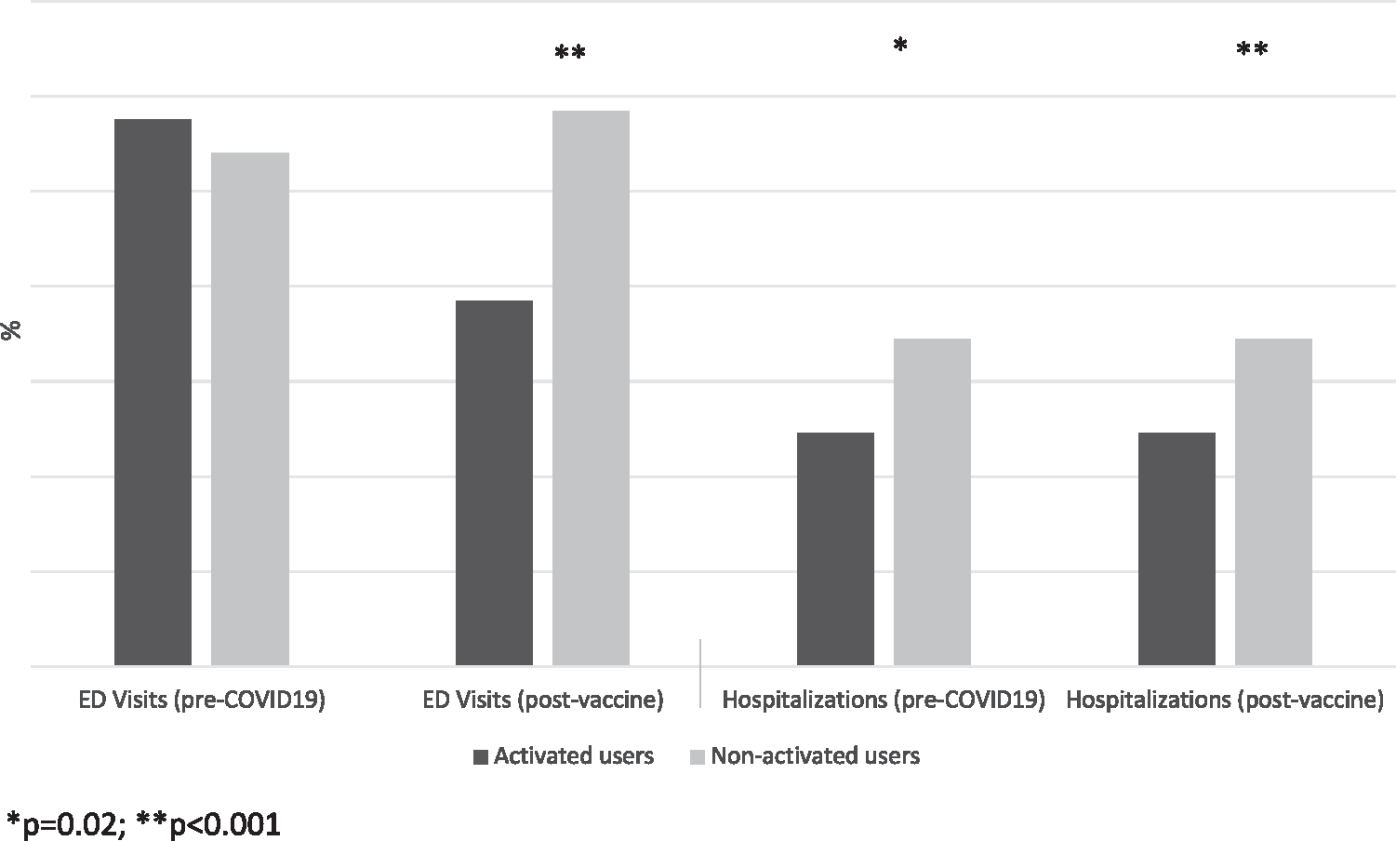
The proportion of asthma-related emergency department (ED) visits and hospitalizations for pediatric patients with asthma by activated versus nonactivated patient portals during pre–COVID-19 (March 19, 2019, to March 19, 2020) and postvaccine (March 19, 2021, to March 19, 2022) time periods.

**TABLE I.** Odds ratios (ORs), unadjusted and adjusted, for demographic characteristics and asthma-related clinical outcomes

Unadjusted				Adjusted			
Outcome: asthma ED visits							

Predictor	OR	95% CI	*P* value*	Predictor	aOR	95% CI	*P* value

Activated	0.59	0.46–0.74	**<.001**	Activated	0.71	0.55–0.93	**.012**
Female vs male	0.87	0.71–1.06	.16	Female vs male	0.85	0.69–1.05	.127
Asian vs White	0.73	0.5–1.07	.023	Asian vs White	0.79	0.53–1.18	.319
Black vs White	1.93	1.36–2.74	**<.001**	Black vs White	1.42	0.96–2.09	**.03**
Hispanic vs White	1.5	1.18–1.92	**<.001**	Hispanic vs White	1.09	0.81–1.46	.115
Other race vs White	0.52	0.39–0.69	**<.001**	Other race vs White	0.55	0.41–0.74	**<.001**
Age (+1 y)	0.97	0.95–0.99	**.005**	Age (+1 y)	0.96	0.94–0.98	**<.001**
Other insurance vs private	0.34	0.12–0.96	.354	Other insurance vs private	0.36	0.13–1.02	.249
Private vs public	0.31	0.25–0.38	**.019**	Private vs public	0.44	0.34–0.57	.267
SVI	1.09	1.05–1.13	**<.001**	SVI	0.91	0.60–1.38	.666
Mild persistent vs mild intermittent	1.49	0.93–2.39	**.007**	Mild persistent vs mild intermittent	1.40	0.87–2.25	**.016**
Moderate persistent vs mild intermittent	3.45	2.42–4.94	**.027**	Moderate persistent vs mild intermittent	3.14	2.18–4.52	**.021**
Severe persistent vs mild intermittent	4.1	1.79–9.37	.13	Severe persistent vs mild intermittent	3.07	1.31–7.22	.340
Unspecified severity vs mild intermittent	4.59	3.53–5.97	**<.001**	Unspecified severity vs mild intermittent	4.02	3.07–5.25	**<.001**
English vs Spanish preferred language	0.41	0.28–0.61	**<.001**	English vs Spanish preferred language	1.01	0.64–1.59	.965
Unknown smoker vs never smoker	0.47	0.32–0.67	**<.001**	Unknown smoker vs never smoker	0.51	0.34–0.75	**<.001**
Current/former smoker vs never smoker	2.86	1.15–7.12	**.002**	Current/former smoker vs never smoker	2.24	0.84–6.01	**.025**

**Outcome: asthma hospitalizations**							

Activated	0.41	0.3–0.54	**<.001**	Activated	0.47	0.34–0.65	**<.001**
Female vs male	0.88	0.67–1.16	.36	Female vs male	0.89	0.67–1.18	.430
Asian vs White	1.07	0.66–1.75	.61	Asian vs White	1.10	0.67–1.82	.379
Black vs White	1.77	1.07–2.92	.03	Black vs White	1.06	0.61–1.85	.520
Hispanic vs White	1.83	1.3–2.56	**<.001**	Hispanic vs White	0.92	0.61–1.38	.900
Other race vs White	1.66	0.44–0.97	**<.001**	Other race vs White	0.66	0.44–0.99	**.017**
Age (+1 y)	0.94	0.92–0.97	**<.001**	Age (+1 y)	0.93	0.90–0.96	**<.001**
Other insurance vs private	1.34	0.62–2.91	**.011**	Other insurance vs private	1.55	0.69–3.52	**.027**
Private vs public	0.25	0.19–0.32	**<.001**	Private vs public	0.40	0.28–0.56	**<.001**
SVI	1.14	1.09–1.19	**<.001**	SVI	1.23	0.71–2.14	.456
Mild persistent vs mild intermittent	1.28	0.7–2.33	**.046**	Mild persistent vs mild intermittent	1.20	0.65–2.21	.143
Moderate persistent vs mild intermittent	3.25	2.11–5.10	**.014**	Moderate persistent vs mild intermittent	2.77	1.77–4.33	**.013**
Severe persistent vs mild intermittent	3.3	1.15–9.49	.275	Severe persistent vs mild intermittent	2.04	0.67–6.28	.714
Unspecified severity vs mild intermittent	2.86	2.05–5.01	**.028**	Unspecified severity vs mild intermittent	2.31	1.64–3.25	.061
English vs Spanish	0.21	0.14–0.32	**<.001**	English vs Spanish	0.55	0.33–0.91	**.021**
Unknown smoker vs never smoker	0.05	0.01–0.21	**<.001**	Unknown smoker vs never smoker	0.05	0.01–0.18	**<.001**
Current/former smoker vs never smoker	2.32	0.69–7.75	**.001**	Current/former smoker vs never smoker	1.79	0.49–6.57	**.004**

Asthma ED visits by language							

Predictor and subgroup	OR	95% CI	*P* value	Predictor and subgroup	aOR	95% CI	*P* value

Activated and Spanish preferred	1.03	0.69–1.54	.878	Activated and Spanish preferred	1.10	0.71–1.72	.663
Activated and English preferred	0.60	0.47–0.75	**<.001**	Activated and English preferred	0.64	0.49–0.84	**.001**

**Asthma hospitalization by language**							

Activated and Spanish preferred	0.68	0.45–1.02	.065	Activated and Spanish preferred	0.72	0.46–1.14	.160
Activated and English preferred	0.27	0.28–0.48	**<.001**	Activated and English preferred	0.40	0.28–0.55	**<.001**

*aOR,* Adjusted odds ratio; *CI,* confidence interval; *ED,* emergency department; *SVI,* Social Vulnerability Index.

* A *P* value of <.05 is considered statistically significant (boldface).

## Clinical Implications

We found that portal use in pediatric patients with asthma was more likely to be by historically advantaged groups, and English-language preferred activated portal users were less likely to experience asthma emergency department visits and hospitalizations than nonusers when controlling for other characteristics.
