# Supplementary material for: Electronic patient portal activation and outcomes among pediatric patients with asthma
Source: J Allergy Clin Immunol Pract. Author manuscript; Available in PMC 2023 Dec 28. (PMC10752757; doi:10.1016/j.jaip.2023.03.019)
Supplement: 1 [file NIHMS1950790-supplement-1.pdf]

ONLINE REPOSITORY

TABLE E1. Demographic characteristics of pediatric patients with asthma whose portals were activated versus nonactivated in the pre–COVID-19 (March 19, 2019, to March 19, 2020) and postvaccine era (March 19, 2021, to March 19, 2022)

	Pre–COVID-19			Post–COVID-19 vaccine		
Variable	Activated (N = 4542, 80%)	Nonactivated (N = 1167, 20%)	*P* value*	Activated (N = 5469, 92%)	Nonactivated (N = 478, 8%)	*P* value
Sex, n (%)			**.001**			.074
Female	1892 (41.7)	426 (36.5)		2243 (41)	176 (36.8)	
Male	2650 (58.3)	741 (63.5)		3226 (59)	302 (63.2)	
Race, n (%)			**<.001**			**<.001**
Asian	549 (12.1)	52 (4.5)		607 (11.1)	18 (3.8)	
Black/African American	321 (7.1)	126 (10.8)		385 (7)	51 (10.7)	
White	1819 (40)	419 (35.9)		2098 (38.4)	175 (36.6)	
Other†	1852 (40.8)	570 (48.8)		2379 (43.5)	234 (49)	
Ethnicity, n (%)			**<.001**			**<.001**
Hispanic/Latino or Latina	964 (21.2)	309 (26.5)		1201 (22)	150 (31.4)	
Not Hispanic/Latino or Latina	2878 (63.4)	582 (49.9)		3301 (60.4)	225 (47.1)	
Unknown/patient refused to answer	700 (15.4)	276 (23.7)		967 (17.7)	103 (21.5)	
Age range (y), n (%)			**.022**			.33
0-4	558 (12.3)	130 (11.1)		806 (14.7)	76 (15.9)	
5-11	2107 (46.4)	503 (43.1)		2341 (42.8)	188 (39.3)	
12-18	1875 (41.3)	534 (45.8)		2320 (42.4)	214 (44.8)	
Insurance type, n (%)			**<.001**			**<.001**
Other	41 (0.9)	24 (2.1)		63 (1.2)	11 (2.3)	
Private	3889 (85.6)	721 (61.8)		4569 (83.5)	247 (51.7)	
Public	607 (13.4)	404 (34.6)		830 (15.2)	217 (45.5)	
SVI‡, mean (n), SD	0.33 (4169), 0.26	0.41 (1054), 0.29	**<.001**	0.35 (4965), 0.27	0.46 (409), 0.3	**<.001**
Specialist, n (%)			.094			.97
Allergy + pulmonology	1447 (31.9)	342 (29.3)		1551 (28.4)	136 (28.5)	
Generalist	3095 (61.8)	825 (70.7)		3918 (71.6)	342 (71.5)	
Severity, n (%)			**<.001**			**<.001**
Mild intermittent	1598 (35.2)	353 (30.3)		2246 (41.1)	152 (31.8)	
Mild persistent	358 (7.9)	83 (7.1)		501 (9.2)	34 (7.1)	
Moderate persistent	435 (9.6)	85 (7.3)		540 (9.9)	55 (11.5)	
Severe persistent	46 (1)	17 (1.5)		49 (0.9)	10 (2.1)	
Unspecified/none of the above	2105 (46.4)	629 (53.9)		2049 (37.5)	221 (46.2)	
Preferred language, n (%)			**<.001**			**<.001**
English	4419 (97.3)	1047 (89.7)		5283 (96.6)	407 (85.1)	
Spanish	93 (2)	95 (8.1)		143 (2.6)	59 (12.3)	
Other‖	30 (0.7)	25 (2.1)		43 (0.8)	12 (2.5)	
Smoking status, n (%)			**<.001**			**<.001**
Current/former smoker	11 (0.9)	22 (0.5)		2 (0.4)	29 (0.5)	
Nonsmoker	867 (74.3)	4102 (93)		353 (73.8)	4696 (85.9)	
Did not assess	289 (24.8)	418 (9.2)		123 (25.7)	744 (13.6)	

*A *P* value of <.05 is considered statistically significant (boldface).
†Includes American Indian/Alaskan Native, Middle Eastern/North African, Native Hawaiian/Pacific Islander, Multiple Races, Patient Refused, Unknown, and Other.
‡SVI, Social Vulnerability Index. This refers to the potential negative effects on communities caused by external stresses on human health. A percentile ranking represents the proportion of counties that are equal to or lower than a county of interest in terms of social vulnerability. For example, an SVI ranking of 0.85 signifies that 85% of counties in the state or nation are less vulnerable than our county of interest and that 15% of counties in the state or nation are more vulnerable.[E1]
‖Includes Arabic, Chinese, Farsi, German, Japanese, Korean, Nepali, Portuguese, Russian, Sign Language, Tagalog, Urdu Pakistan, Vietnamese, Unknown, and Other.

TABLE E2. Portal use types among activated users in the pre–COVID-19 versus postvaccine era

Portal use types among activated users	Pre–COVID-19 Dates: March 19, 2019, to March 19, 2020 (N = 4542)	Postvaccine Dates: March 19, 2021, to March 19, 2022 (N = 5469)	*P* value*
Portal use by proxy	3340 (73.5)	4504 (82.4)	**<.001**
Messages with physician/practice			
Read message	3112 (68.5)	4228 (77.3)	**<.001**
Wrote message	1641 (36.1)	2068 (37.8)	**<.001**
Appointments			
Requested	523 (11.5)	475 (8.7)	**<.001**
Medications			
Read	1977 (43.5)	3504 (64.1)	**<.001**
Updated	307 (6.8)	754 (13.8)	**<.001**
Health summary read			
Read visit summary	37 (0.8)	30 (0.6)	.13
Lab test results			
Read details	1725 (38.0)	3263 (59.7)	**<.001**

P values from McNemar's test on paired data. Data are presented as n (%).
*A *P* value of <.05 is considered statistically significant (boldface).

How SVI was applied to our population

1. Our institution uses a geocoding application (ESRI ArcMap Desktop 10.x) to calculate the latitude and longitude for each University of California, Los Angeles patient's address with the help of a geocoder file from the vendor.
2. The census tract and block group (2021 TIGER-Line Shapefiles: Block Groups) is calculated for each address using the TIGER files from the US Census website.
3. Match this information to SVI datasets from the Centers for Disease Control website (https://www.atsdr.cdc.gov/placeandhealth/svi/at-a-glance_svi.html), which uses data from the 2018 5-year American Community Survey to calculate their indexes.
4. We keep the SVI related to the patient's most current address on record and that is what is used.

REFERENCE

E1. Localio AM, Klusaritz H, Morales KH, Ruggieri DG, Han X, Apter AJ. Primary language and the electronic health record patient portal: Barriers to use among Spanish-speaking adults with asthma. J Asthma 2022;59:2081-90.
